# Growth Stimulation of Durum Wheat and Common Buckwheat by Non-Thermal Atmospheric Pressure Plasma

**DOI:** 10.3390/plants12244172

**Published:** 2023-12-15

**Authors:** Barbora Tunklová, Božena Šerá, Petra Šrámková, Sandra Ďurčányová, Michal Šerý, Dušan Kováčik, Anna Zahoranová, František Hnilička

**Affiliations:** 1 Department of Botany and Plant Physiology, Faculty of Agrobiology, Food and Natural Resources, Czech University of Life Sciences Prague, Kamýcká 129, 165 00 Prague, Czech Republic; tunklova@af.czu.cz (B.T.); hnilicka@af.czu.cz (F.H.); 2 Department of Environmental Ecology and Landscape Management, Faculty of Natural Sciences, Comenius University in Bratislava, Ilkovičova 6, 842 15 Bratislava, Slovakia; 3 Department of Experimental Physics, Faculty of Mathematics, Physics and Informatics, Comenius University in Bratislava, Mlynská Dolina, 842 48 Bratislava, Slovakia; petra.sramkova@fmph.uniba.sk (P.Š.); sandra.durcanyova@fmph.uniba.sk (S.Ď.); zahoranova1@uniba.sk (A.Z.); 4 Department of Physics, Faculty of Education, University of South Bohemia, Jeronýmova 10, 371 15 České Budějovice, Czech Republic; kyklop@pf.jcu.cz

**Keywords:** *Fagopyrum esculentum*, low-temperature plasma, plasma treatment, seed, seedling, *Triticum durum*

## Abstract

The grains of durum wheat (*Triticum durum* Desf.) and achenes of common buckwheat (*Fagopyrum esculentum* Moench) were tested after treatment with two sources of non-thermal atmospheric pressure plasma (DCSBD, MSDBD) with different treatment times (0, 3, 5, 10, 20, 30, and 40 s). The effect of these treatments was monitored with regard to the seed surface diagnostics (water contact angle—WCA, chemical changes by Fourier transform infrared spectroscopy—FTIR); twenty parameters associated with germination and initial seed growth were monitored. A study of the wettability confirmed a decrease in WCA values indicating an increase in surface energy and hydrophilicity depending on the type of seed, plasma source, and treatment time. Surface analysis by attenuated total reflectance FTIR (ATR-FTIR) showed no obvious changes in the chemical bonds on the surface of the plasma-treated seeds, which confirms the non-destructive effect of the plasma on the chemical composition of the seed shell. A multivariate analysis of the data showed many positive trends (not statistically significant) in germination and initial growth parameters. The repeated results for germination rate and root/shoot dry matter ratio indicate the tendency of plants to invest in underground organs. Durum wheat required longer treatment times with non-thermal plasma (10 s, 20 s) for germination and early growth, whereas buckwheat required shorter times (5 s, 10 s). The responses of durum wheat grains to the two non-thermal plasma sources used were equal. In contrast, the responses of buckwheat achenes were more favorable to MSDBD treatment than to DCSBD.

## 1. Introduction

The impacts of climate change and the need to respond to these changes in agriculture do not only concern areas in which negative changes have already occurred but are also relevant in temperate climate areas [1,2,3]. These changes are manifested mainly by the more pronounced instability of the moisture and temperature conditions of agricultural land. Thus, the characteristics of individual production areas are gradually changing, and so is their suitability for growing traditional agricultural crops [4]. The recommended non-conventional crops of Europe include, for example, durum wheat (*Triticum durum* Desf.) and common buckwheat (*Fagopyrum esculentum* Moench) as cereal and pseudocereal, respectively [5,6,7,8,9].

Compared to common wheat (*Triticum aestivum* L.), durum wheat is characterized by stronger gluten, a yellow grain color, lower glycemic index, and longer durability, which are all essential properties to make pasta [10]. Durum wheat originated from emmer wheat (*Triticum dicoccon* L.) [11]. The global Mediterranean-like climates where durum wheat is grown are hot-spots of climate change, where the temperature is warming faster than in other world regions [12]. Therefore, researchers are coming up with new insights into genetic resources and suggestions for better cultivation practices of durum wheat in temperate regions [13,14,15,16]. Germination and initial growth are among the most important factors monitored.

There is an increased market demand for gluten-free products to help promote human health. Buckwheat is gluten free, but contains rutin and lecithin, so it provides health benefits beyond basic nutrition [17,18]. Buckwheat has a wide range of agronomic and health benefits that make it a promising pseudocereal for sustainable agricultural production [19]. Due to significant differences in environmental conditions (during germination and early plant establishment), differences in the growth, yield, and quality of buckwheat are often documented [20,21,22]. Differences in growth are probably related to the sowing date, duration of the pre-flowering period, and biosynthesis of assimilates [19].

Some stimulation effects on buckwheat achenes can help in non-optimal sowing dates. Specifically, they can promote a more uniform rate of germination and emergence of seeds in a field. To improve seed germination and growth under changing environmental conditions, techniques such as chemical [23], physical [24], and biological [25] treatments are being developed. Besides the use of harmful fertilizers, physical methods such as electromagnetic waves, ionizing radiation, ultrasound, or non-thermal plasma offer an ecological, non-invasive, and simple manner to enhance seed germination and growth. Among other agents, non-thermal plasma (NTP) is attracting increasing interest in agriculture [26,27] as it is suitable not only for improving seed germination but also for microbial inactivation on seed surfaces [28,29]. Moreover, plasma treatment may induce the formation of active compounds in the sprouting seeds [30], thereby increasing the nutrient content of the plants and extending the shelf life of fruits and vegetables in the post-harvest phase [31,32]. NTP applications have advantages over conventional treatments due to the short treatment time, easy accessibility, and low temperatures during operation. In addition, NTP is very gentle and therefore only affects a thin layer of the treated substrates to avoid mechanical damage to seeds and plants. Plasma sources primarily used for seed treatment include plasma jet, gliding arc, dielectric barrier discharge, and corona discharge in various configurations [33], which are able generate NTP at atmospheric pressure in ambient air. The suitability of these sources is related to their high reactivity without the need for low pressures and the possibility to feasibly transfer their configuration from a laboratory to an industrial scale.

Only a few articles have dealt with the plasma treatment of buckwheat and durum wheat species. Guiyun et al. [34] employed the dielectric barrier discharge plasma source to treat buckwheat at different treatment times and discharge powers. They observed an increase in the concentration of γ-aminobutyric acid, known as GABA, in sprouted buckwheat after plasma treatment. Another study describes the effect of plasma treatment on six durum wheat varieties in relation to germination, antifungal activity, and plant growth [35]. Cold plasma was generated using a low-pressure radio frequency system with oxygen as the feed gas to test the decontamination efficacy of different fungi-colonizing buckwheat grains [36].

Both buckwheat and durum wheat are considered promising and important crops for non-conventional agriculture in Europe [5,7,9]. We therefore decided to test the responses of these crops to NTP, which improves germination and initial plant growth, including the effect on the root system [26,27,37,38]. In the present work, the authors have studied the effect of NTP generated at atmospheric pressure by a diffuse coplanar surface barrier discharge (DCSBD) and a multi-hollow surface dielectric barrier discharge (MSDBD) on durum wheat caryopses and buckwheat achenes. The main scientific aims of the study were (i) to evaluate the effect of two NTP sources using different treatment times on grains of durum wheat and achenes of buckwheat, (ii) to describe fundamental NTP effects on grain/achene surface properties, and (iii) to estimate the optimal NTP treatment in relation to the seed germination and early growth of seedlings.

## 2. Results

### 2.1. Fruit Surface Diagnostic

**Figure 1:**
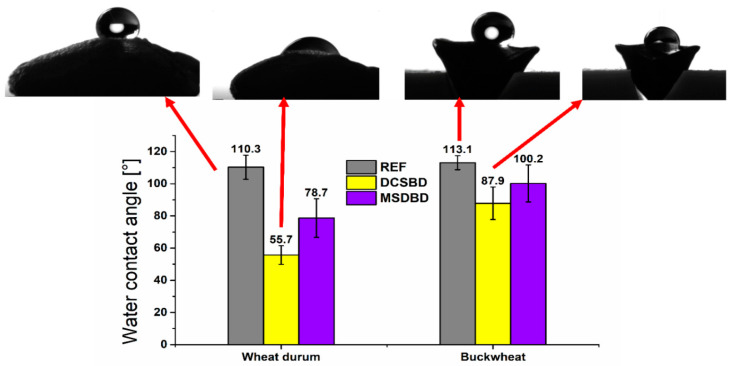
Effect of NTP treatment with DCSBD and MSDBD on the WCA measured on durum wheat grains and buckwheat achenes at optimal treatment times (buckwheat: DCSBD = 10 s, MSDBD = 5 s; durum wheat: DCSBD = 20 s, MSDBD = 30 s). The droplet images are assigned to resulting WCA values in graphs using arrows.

Fruits/seeds are naturally protected by a lipid layer on their surface, causing the hydrophobic character of the seed shell. Plasma treatment primarily induces the oxidation of this layer, which is accompanied by hydrophilization and an improvement in the wettability of the seed surface. This process was monitored by water contact angle (WCA) measurements and the results are shown in Figure 1. The model seeds were analyzed at only one selected plasma treatment time determined as the optimal plasma treatment time with both plasma discharges. Considering the results of germination and initial seed growth parameters, the optimal treatment time for durum wheat with DCSBD was determined as 20 s and with MSDBD as 30 s. Similarly, in the case of buckwheat, the optimal treatment time by DCSBD was set to 10 s and by MSDBD to 5 s. Treatment of durum wheat with DCSBD resulted in a decrease in WCA from 110.3° to 55.8° (20 s) and with MSDBD to 78.7° (30 s). For buckwheat, the determined optimal plasma treatment times were much lower, so the decrease in WCA was not as pronounced. The DCSBD treatment caused a decrease in WCA from 113.1° to 87.9°, whereas the MSDBD treatment only caused a reduction to 100.2°. Considering the different shapes of the seeds, the efficiency and uniformity of the plasma treatment also depend on their ability to move homogeneously through the plasma during the treatment. Since wheat has an elongated round shape, the grains were continuously rotated on the DCSBD ceramic, resulting in a more homogeneous treatment, which was reflected in lower deviations in the achieved WCA value (55.7° ± 5.8°). In comparison, the deviation of the WCA value in buckwheat treated with DCSBD in 10 s was 10.1° due to the triangular shape of buckwheat, which limits the homogeneous motion of the seeds in the lower time intervals.

**Figure 2:**
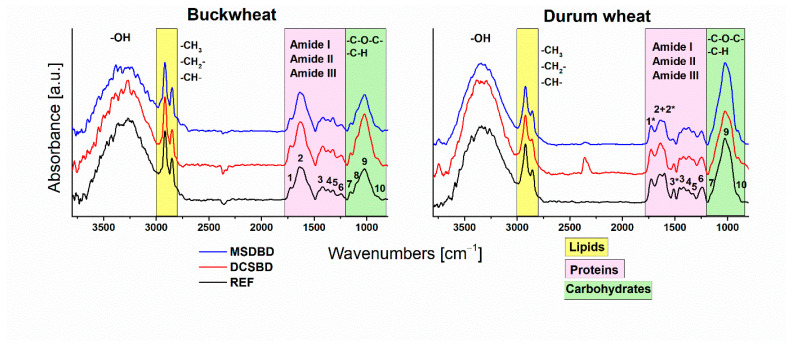
ATR-FTIR spectra of buckwheat and wheat durum before (control sample) and after plasma treatment with DCSBD and MSDBD in ambient air at optimal treatment times (buckwheat: DCSBD = 10 s, MSDBD = 5 s; durum wheat: DCSBD = 20 s, MSDBD = 30 s). Peaks of characteristic functional groups are marked by numbers above the peak (assignment of numbers is in Table 1). The * at the number in spectrum of durum wheat marks the extra functional groups present only in this spectrum (to preserve the same numbering in both spectra).

Different seeds consist of different nutrients (including protein, carbohydrates, and lipids) in variable ratios. ATR-FTIR spectroscopy has been used to describe the biochemical composition of certain seeds and to monitor changes in composition after plasma treatment. Figure 2 shows the FTIR spectra before and after plasma treatment of certain seeds. In durum wheat and buckwheat, the cereals and pseudocereals in our study, the main components are carbohydrates, namely starch and dietary fiber along with proteins and lipids. Especially, whole cereal grains are considered a rich source of dietary fiber (cellulose, hemicellulose, lignin), which is mainly concentrated in the upper layers of the seeds (pericarp, hull) which is also the subject of our study since plasma directly affects only the thin layer of the seed’s surface. According to the study by [39], the hull of various buckwheat varieties consists of about 90% carbohydrates, including starch (~2.5%) and dietary fiber (~85%). In comparison, the husk of durum wheat contains ~53% dietary fiber and ~30% starch [40]. Since we only treated the surface of the seeds, the recorded FTIR spectra reflected the composition of the fewmicrons-thin upper layer. A strong, broad peak in the range of 3000–3700 cm^−1^, which is assigned to the –O–H stretching vibrations, can originate from cellulose, hemicellulose, and lignin, as well as starch. Two further peaks in the range from 2800 to 3000 cm^−1^ represent the symmetrical and asymmetrical C-H stretching vibrations of the CH_3_, CH_2_, and CH groups, which are normally assigned to lipids due to the long aliphatic chain but also occur in the spectra of the polysaccharides mentioned. Then, in the fingerprint range of wavelengths from 800 to 1800 cm^−1^, we marked the peaks with the numbers 1–10. In the spectra of both studied seeds, these ten characteristic bands indicate the presence of cellulose, hemicellulose, starch, and also lignin [41,42]. Table 1 summarizes the absorption ranges of the corresponding peaks as well as their assignment and possible sources. In this range, the vibrations of various bonds belonging to the polysaccharides dominate; however, the characteristic range for Amides I, II, and III may overlap due to the presence of proteins in the shell. No obvious changes were observed in the FTIR spectra after plasma treatment, which underlines the non-invasive effect of plasma on the bulk of the treated material. Since the penetration depth of the ATR-FTIR technique is only a few micrometers, we can conclude that the plasma treatment did not significantly change the chemical composition of the seed surface.

### 2.2. Germination and Early Growth

The measured parameters for both plants treated with two types of NTP are shown in Table 2 and Table 3. No parameters showed any significant positive changes (*p* < 0.05) in the treated seeds compared to the control sets. Statistical analyses found many significant differences between the control samples and samples after 40 s exposition of NTP with negative effects on the germination and early growth (Table 2 and Table 3).

The same positive trends were found (not statistical level, *p* < 0.05) in many measured parameters. Germination increased especially in buckwheat (treatment time 5 s, MSDBD plasma), where on the 6th day it reached a value of 138% compared to the control (treated samples 60.7%, control samples 44.0%). The germination of durum wheat seeds, on the other hand, was balanced with regard to all treatment times and the types of equipment used (both in Table 2). Other positive trends (not statistical level, *p* < 0.05) were found in buckwheat, always after MSDBD plasma treatment: with exposure for 5 s (difference compared to control more than 30%)—germination index, seedling vigor index II, seedling vigor index III; with exposure of 10 s (difference from control 124%)—R/S_length; with exposure of 40 s (difference from control 300%)—R/S_dry_weight. These trends show high variability; therefore, no statistical difference was recorded at *p* < 0.05 (Table 2).

Table 4 presents the sum of positive trends in germination and early growth characteristics in the presented experiment. The treatment of buckwheat and durum wheat by non-thermal plasma has a positive effect on germination parameters at certain plasma exposure times which differ between seed types and plasma sources. The optimal times for non-thermal plasma treatment were 5 s and 10 s for buckwheat; for durum wheat, the times were 20 s and 10 s. If we compare the two plasma sources used, then MSDBD plasma was more suitable than DCSBD plasma for the treatment in our experiment. If we go to the level of individual plants, the MSDBD plasma treatment was more suitable for buckwheat and the DCSBD plasma treatment for durum wheat.

**Figure 3:**
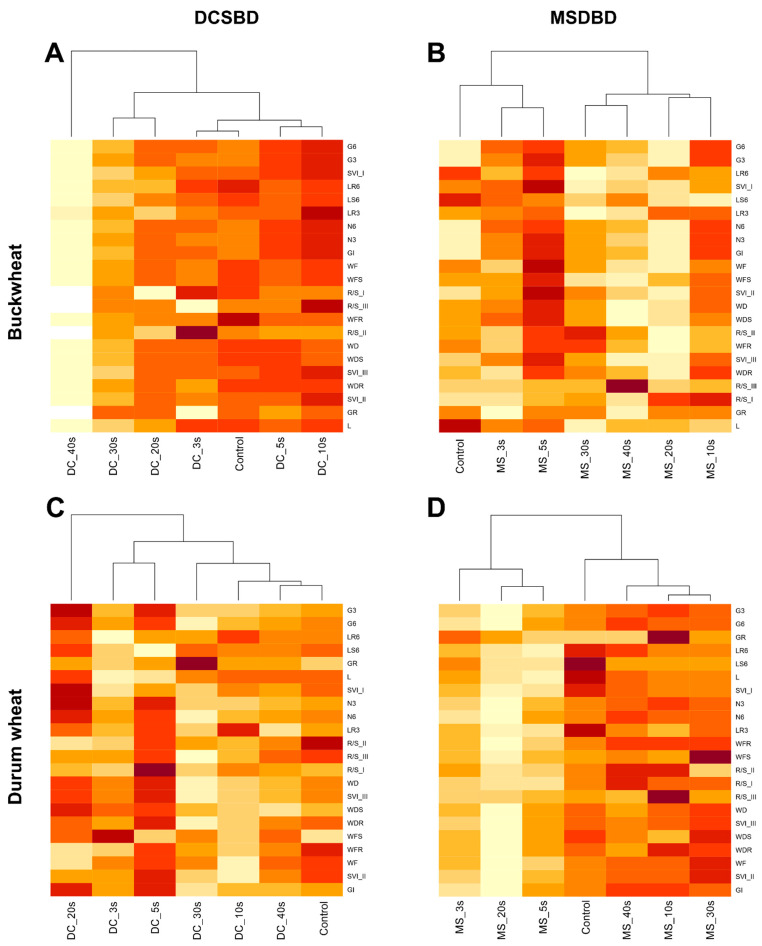
Heatmap histogram correlation between measured characteristics and used non-thermal plasma treatments. Two plasma treatments (DCSBD and MSDBD) in various time expositions on buckwheat and durum wheat are presented. Subfigures (**A**–**D**) correspond to individual combinations between NTP type and plant species. The abbreviations of the characteristics are explained in the 4.5. Germination and Early Growth.

According to heatmap correlations, two different clusters were formed between the treatments (Figure 3); in the MSDBD plasma treatment (buckwheat and durum wheat) and in durum wheat treated with DCSBD plasma, the structures of the generated branches are the same, but they always contain different treatment times (Figure 3B–D). For buckwheat treated with DCSBD plasma, one treatment cluster is 40 s and the other cluster consists of all other treatment times (Figure 3A). For buckwheat treated with DCSBD plasma and durum wheat treated with MSDBD plasma, responses to shorter treatment times (including 0 s as a control) are always in one cluster and responses to higher times are in the second cluster (Figure 3B,C). For durum wheat treated with MSDBD plasma, the longer times including the response of the control set are in the first cluster (0 s, 30 s, 40 s) and the lower treatment times are in the second cluster (3 s–20 s; Figure 3D).

**Figure 4:**
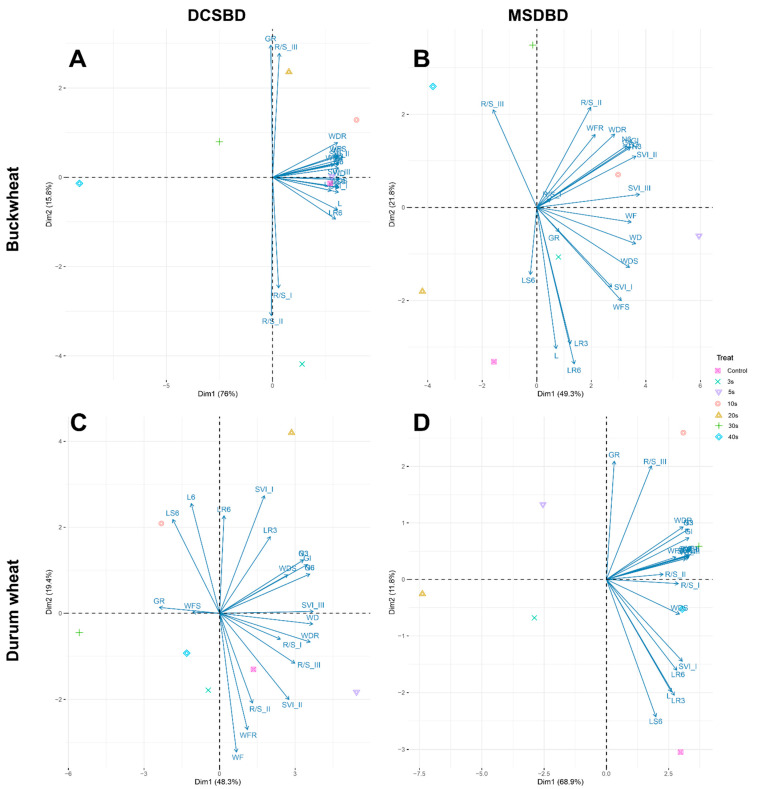
Plots of Principal Component Analysis (PCA) on measured characteristics in treated buckwheat and durum wheat by non-thermal plasma treatments (DCSBD and MSDBD) after various time expositions. Subfigures (**A**–**D**) correspond to individual combinations between NTP type and plant species. The abbreviations of the characteristics are explained in the 4.5. Germination and Early Growth.

The outputs of the Principal Component Analyses (PCA) for both plants in relation to the two types of NTP treatments are presented in Figure 4. The treatments (plasma exposure times) are spread in the whole dataset in different ways in relation to the plants (buckwheat and durum wheat) and to the NTP treatment (DCSBD and MSDBD). In PCA with buckwheat treated with DCSBD plasma, GR and R/S_III are opposite to the characteristics indicating the length and weight of live biomass (LR, LS, WFR, WFS) and the first two components described 91.8% of the overall variance (which is the biggest of all PCAs calculated; Figure 4A). In buckwheat treated with MSDBD plasma, only R/S_III is in opposition to the six characteristics (GR, LR3, LR6, L6, SVI_I, and WFS) in PCA (Figure 4B).

The PCA output for durum wheat treated with DCSBD plasma revealed that the first two components described 67.7% of the overall variance (which is the least of all PCAs calculated) and the projected characteristics are relatively spread over the area (Figure 4C). Here, GR lies against the characteristics representing the weight of dry biomass (SVI_III, WD, WDR) and at the same time the length characteristics (L6, LS6, LR6) lie against the characteristics representing the weight of fresh biomass (SVI_II, WF, WFR, R/S_II; Figure 4C). In PCA with buckwheat treated with DCSBD plasma, GR and R/S_III are opposite to the five characteristics indicating length (LS6, L6, LR3, LR6, SVI_I; Figure 4D). 

## 3. Discussion

For the plasma treatment, we used and compared two types of plasma sources (DCSBD and MSDBD). The first one, DCSBD, has already been widely used and studied in regard to seed surface treatment [29,43,44], while MSDBD has only been used in a few studies so far [45,46]. Both plasma sources can use ambient air as the working gas (but they also work with other gases such as nitrogen, oxygen, and their mixtures). The main difference between the plasma sources used is that with the DCSBD plasma treatment, the seeds move in the active plasma zone, which has an effective thickness of 0.3 mm. In the case of MSDBD, the active plasma species flow through the holes to a longer distance, and the seeds are more homogenously surrounded by active particles. On the other hand, the DCSBD plasma source has a larger active plasma area (8 × 20) cm^2^, while MSDBD has an active area of (19 × 18) mm^2^. The advantage of the MSDBD is its portability.

We studied changes in surface properties after plasma treatment for the case of optimal times of plasma treatment determined on the basis of germination and early growth results. The optimal treatment time for durum wheat with DCSBD was established as 20 s and with MSDBD as 30 s (Table 2 and Table 3). Similarly, in the case of buckwheat, the optimal treatment time was established as 10 s with DCSBD and 5 s with MSDBD (Table 2 and Table 3). As shown by the decrease in WCA values (Figure 1), we recorded a more significant decrease for durum wheat, which can be attributed to a longer treatment time. The shape of the seeds had an influence on the homogeneity of the treatment, expressed by the deviation from the mean values. However, in both cases, the treated seeds gained hydrophilic properties, resulting in better water absorption and better conditions for germination initiation.

Similarly, for the same optimal treatment times of both types of seeds, we performed surface diagnostics using ATR-FTIR. The results (Figure 2, Table 1) clarified the surface composition of the seed coat (mainly proteins, lipids, and carbohydrates, originating from lignin, cellulose, and hemicellulose), and no obvious changes were observed in the ATR-FTIR spectra after plasma treatment. This result confirms that the plasma has no destructive effect on the surface of the seeds. Since we monitor the composition to a depth of several micrometers when using ATR-FTIR, the changes due to the interaction of plasma particles with the surface may be evident at the nanometer level.

In general, plasma stimulation of plants should lead to a better resistance to drought and environmental stresses, as well as diseases and pests [27,47]. We found that the DCSBD and MSDBD plasma treatments affected GR and R/S_III in both tested plants, which are important traits for good germination and for increased biomass investment in roots. Strengthening the roots at the expense of the above-ground parts of the seedling can help with drought [27,47]. These stimulatory effects applied to buckwheat achenes or durum wheat grains can positively influence the germination and growth of plants in cases of a sub-optimal sowing date or lack of water in the soil.

New durum wheat cultivars are reported to have thermal growth requirements similar to common wheat and it is reported that the reproductive phase should occur at higher temperatures. The xerophytic character of durum wheat results in their good resistance to soil water deficit, while water stress tolerance is the result of the cumulative effect of various characteristics and physiological processes [48,49,50]. Our results did not show any negative effect on the germination and initial growth of durum wheat (Table 2 and Table 3). Moreover, these grains tended to respond better to longer exposure times of NTP (30 s, 40 s) than to shorter times (Figure 3). Longer exposure times of NTP will heat the grains to a higher temperature. Our results indicate the need for durum wheat to be stimulated by higher temperatures before self-germination. Also, it is probably very important which cultivar of durum wheat is used, as found by Nedyalkova et al. [35] when studying the effect of *Fusarium graminearum* on the growth of six durum wheat cultivars. We can that summarize if the drying of agricultural land in Europe were to progress, perhaps the treatment of grains with NTP could make durum wheat possible for use in non-conventional cultivation.

Regarding buckwheat, its achenes do not show uniform germination and initial growth [20,21,22]. Buckwheat seeds have been tested several times in the past with NTP, e.g., [51,52,53]. In previous experiments [51], it was found that buckwheat is very sensitive to plasma treatment. In this experiment, it was found (in agreement with the previous one) that buckwheat requires very low exposure times to NTP for germination and initial growth. The strengthening of the GR and R/S_III characteristics by the application of NTP (Table 2 and Table 3, Figure 4) that we found could help in the variability of field emergence and in better rooting of seedlings. Ivankov et al. [52] studied the effects of pre-sowing achene treatments of cold plasma and electromagnetic field on the agricultural performance of two buckwheat cultivars. Although the percentage of seedlings that emerged under field conditions was reduced, the achene treatment significantly improved growth parameters and the overall yield (the mass of achenes collected per plant was significantly higher in both cultivars compared to the control). The authors summarized that both types of treatment are suitable for agriculture.

Everything mentioned here strongly supports the idea that NTP treatment of both durum wheat (a promising cereal for Central Europe) and common buckwheat (a nutritionally preferred pseudocereal) has great potential for use in non-conventional agriculture.

## 4. Materials and Methods

### 4.1. Description of Plasma Sources

**Figure 5:**
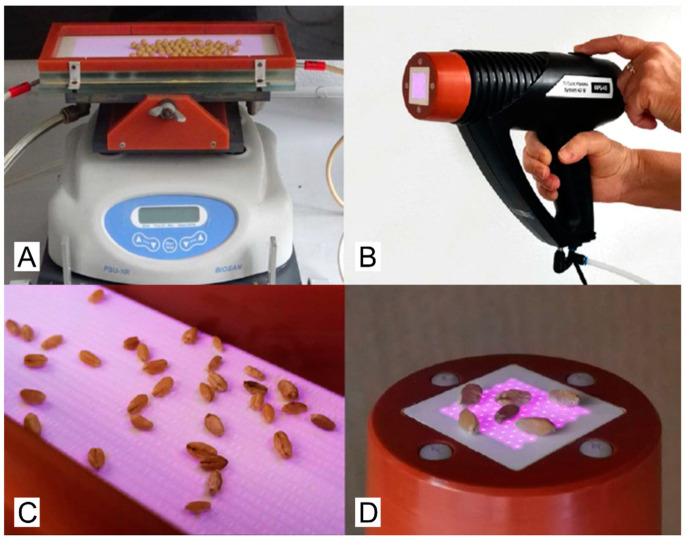
Photo of the DCSBD (**A**) and MSDBD (**B**) plasma source and view of the location of the seeds in the plasma area (**C**,**D**).

Non-thermal plasma (NTP) was generated using two types of dielectric barrier discharges, which differ in their geometric arrangement of electrodes, electrical supply, and active plasma surface area. Both plasma sources allow us to produce low-temperature plasma at atmospheric pressure in ambient air. Both sources of NTP are shown in Figure 5, followed by a description of these sources.

The first was the so-called Diffuse Coplanar Surface Barrier Discharge (DCSBD), which is characterized by its coplanar arrangement of parallel strip-line silver electrodes placed under a ceramic surface (Al_2_O_3_, 96% purity). The discharge is powered by 15 kHz sinusoidal high voltage with an amplitude of up to 20 kV peak-to-peak using the HV generator VF 700 (Lifetech Ltd., Brno, Czech Republic). The DCSBD generates a thin layer (∼0.3 mm) of macroscopically homogeneous plasma on the surface of a ceramic plate, with an active plasma area of (20 × 8) cm^2^. The continuous operation of DCSBD at an input power of 400 W is enabled by an effective cooling and insulating oil system. During seed treatment, the DCSBD is placed on an orbital shaker PSU-10i (BIOSAN, Riga, Latvia) to ensure the rotational motion of the seeds. The detailed characteristics of the DCSBD plasma source were reported in [54].

The second studied plasma source was the Multi-hollow Surface Dielectric Barrier Discharge (MSDBD), which generates NTP in a similar manner to DCSBD and additionally enables the use of flowing gas. We used a commercial RPS30 multi-hollow device (ROPLASS, Brno, Czech Republic). The electrode system consists of two-plane parallel electrodes at a mutual distance of 0.5 mm, fully embedded in ceramic and perforated with 105 holes (Kyocera, Sapporo, Japan). The discharge is generated by sinusoidal high voltage with an amplitude of 13 kV (peak-to-peak) and a frequency of approximately 27 kHz. The total active area on which the plasma is generated is (18 × 19) mm^2^. The advantage of MSDBD over other types of dielectric barrier discharges is that it can operate in gas flow mode (up to 20 lpm). The adjustable gas flow enables better transfer of active plasma species further from the ceramic surface and thus a more effective interaction with the surface of treated material. A more detailed description of MSDBD can be found in previous publications [38].

### 4.2. Plant Material

Cereal grains of durum wheat (*Triticum durum* Desf., variety RU-JH-2022) and pseudocereal achenes of common buckwheat *(Fagopyrum esculentum* Moench, variety Zoe) were obtained from commercial suppliers in the Czech Republic. The weights of a thousand grains or achenes were 52 g or 24 g; seed germinations were 89% and 44% for durum wheat and buckwheat, respectively. Only ripe, intact, surface-untreated (unpeeled), and surface-correct fruits were used in the experiment. One hundred and fifty fruits (grains or achenes) per plant species were used for each treatment (different plasma exposure times) and for reference samples. For simplicity, we usually refer to these fruits (grains and achenes) as seeds in this text.

### 4.3. Plasma Treatment

The DCSBD plasma treatment of cereal grains and pseudocereal achenes was carried out in ambient air at exposure times of 3, 5, 10, 20, 30, and 40 s. The fruits were placed into the plasma layer on top of the ceramic plate and rotated at a rotary speed of 330 rpm. The movement in the discharge area ensured a homogeneous plasma treatment of their surface. The input power of the DCSBD during the treatment was kept at 400 W.

In the case of plasma treatment using the MSDBD plasma source, the grains were also placed directly on top of the discharge surface, surrounded by a plastic enclosure, while the device was rotating horizontally so that the fruits were in motion. Ambient air was fed with a compressor using an RED-Y flow meter (Vögtlin Instruments AG, Muttenz, Switzerland) with a defined constant flow rate of 9 lpm to the plasma source. The exposure times were 3, 5, 10, 20, 30, and 40 s and the input power of the MSDBD during the treatment was kept at 30 W.

### 4.4. Seed Surface Diagnostics

Changes in wettability were monitored by measuring the water contact angle (WCA) on the fruit surface using the Drop Shape Analyser DSA30 (KRŰSS, Hamburg, Germany) and DSA1 software (version V1.92-03). A water drop of 2 mL volume was measured on 12 fruits for each species and each treatment, with the highest and lowest values removed before evaluation. The resulting WCA values were calculated as the average contact angles of 10 droplets for both species. For buckwheat, it was necessary to place the achenes in a suitable holder due to their triangular shape.

Chemical changes on the surface of the fruits for both species were investigated by ATR-FTIR spectroscopy. ATR-FTIR spectra were measured using Bruker Vector 22 spectrometer (Bruker, Berlin, Germany) with an additional accessory, Pike MIRacle^TM^ (with ZnSe crystal; PIKE Technologies, Madison, USA). Spectra were recorded with a resolution of 4 cm^−1^ in the range 4000–600 cm^−1^ with 30 scans for each sample and analyzed with OMNIC software (version 8.0, Thermo scientific, Waltham, MA USA) using baseline correction (linear algorithm) and smoothing (Savitzky-Golay algorithm) of the spectral lines.

### 4.5. Germination and Early Growth

Thirty fruits from one plant species, 1× KA0/80 filter papers, and 7 mL of distilled water were used per one plastic Petri dish of 120 mm in diameter. All experiments were repeated 5× in parallel (5 Petri dishes for one treatment); thus, each treatment and control variant contained 150 seeds. All Petri dishes were kept in a growing box in the dark, temperature approx. 25 °C, 24 h/day. Growth cultivation lasted 6 days.

The number of germinating seeds, shoot length (LS3, LS6), and root length (LR3, LR6) were measured on the 3rd and 6th day of cultivation. Fresh weight of shoot (WFS) and fresh weight of root (WFR) were measured on the 6th day of cultivation. Then, shoots and roots were dried separately at 80 °C for 24 h and the weight of the dried shoots (WDS) and roots (WDR) were determined.

Seed germination (G3, G6) was calculated as a percentage proportion between the number of germinating seeds and the number of seeds used in the germination test. Germination rate (GR) and germination index were calculated according to [55]. The seedling length (L) was obtained as the sum of the length of root (LR6) and length of shoot (LS6) for each seedling; the fresh (WF) and dried weight of seedling (WD) were obtained from WDS and WDR.

Root/shoot ratios were calculated from length (R/S_L), fresh weight (R/S_FW), and from dried weight (R/S_DW) of root and shoot (obtained on the 6th day of cultivation). Three seedling vitality indexes were determined as the product of seed germination and seedling length (SVI_I), seed germination and seedling fresh weight (SVI_II), and seed germination and seedling dry weight (SVI_III). The methodical approach to the used parameters of seed germination and early growth seedlings is described in more detail in [55].

### 4.6. Data Analyses

Statistical analysis of the logarithmically transformed (y = log(x)) data was performed in three steps. All obtained characteristics from both plants were considered as explanatory (active) and treatment with both NTPs at different times as explanatory (supplementary). Each statistical analysis was calculated for each plant (buckwheat, durum wheat) and for each type of plasma source (DCSBD, MSDBD) separately; all calculations were at the 0.05 significance level.

Firstly, analysis of variance (ANOVA) for all obtained characteristics was performed using STATISTICA software (Statistica 13, StatSoft Inc., Tulsa, OK, USA). One-way analysis of variance (ANOVA) was used to evaluate the influence of the NTP treatments on the measured characteristics. Detailed testing of the experimental variances was conducted using the Tukey HSD test for multiple comparisons. The data from the Tukey HSD test are presented in detail; significant differences are indicated by different letters.

Secondly, in the R project (version 4.3.1), the ggplot2; ComplexHeatmap; corrplot; and FactoMineR packages were used to plot the PCA and heatmap histogram correlation. Pearson’s correlation coefficients were used to assess associations between characteristics and treatments. Four cluster analyses were obtained; they present the relationships between all characteristics of seed germination and early growth (20 characters for both plants) and NTP treatment (two different treatments, seven treatment times). Thirdly, a principal component analysis (PCA) was performed to reveal the interrelationship of the studied characteristics with respect to the treatments used (same design as for heatmap histogram correlation).

## 5. Conclusions

In this project, durum wheat and buckwheat were tested as important representatives of non-conventional crops of Europe, subjected to treatment by two different sources of non-thermal plasma (DCSBD, MSDBD) with different treatment times.

Using surface diagnostic methods (WCA measurement and ATR-FTIR analysis), we can detect changes in surface properties after plasma treatment. The WCA measurement showed an increase in surface energy, which means an increase in the hydrophilicity of the seed surface and leads to better wettability and water absorption. Surface analysis using ATR-FTIR pointed out the negligible changes in surface chemical composition of the seeds, indicating the not-destructive character of used non-thermal plasma.

It was found that plasma treatment increased many values of the observed parameters describing germination and initial growth, but not at a statistically significant level (ANOVA, *p* < 0.05). Many trends in the positive growth of the tested plants were detected by multivariate methods. Buckwheat tends to germinate and grow best after MSDBD plasma treatment for 5 s; treatment with DCSBD or MSDBD plasma for 10 s was also positive. It was different for durum wheat, which germinated and grew in a wider spectrum of treatments of both plasma sources, preferably with a processing time of 10 s and 20 s. According to the twenty analyzed germination and initial growth parameters, the germination rate (GR) and root/shoot dry matter ratio (R/S_III) were determined as the two main traits. These traits determine good germination and also the preference of investing in the roots over the above-ground parts. Plasma stimulation for better germination and root investment can help young durum wheat and common buckwheat plants during periods of water shortage in the soil.

## Figures and Tables

**Table 1:** Assignment of absorption bands in FTIR spectra of buckwheat and durum wheat. * marks extra functional groups present only in the durum wheat spectrum (to preserve the same numbering in both spectra).

Peak	Absorption Band [cm^−1^]	Assignments	Source of Peak
1 + 1 *	1760–1700	C=O stretching vibrations of carbonyl, carboxyl groups	lignin, hemicellulose
2 + 2 *	1670–1530	Amide I, Amide II (C=O, N–H, C–N vibrations), aromatic ring vibrations	proteins, lignin
3	1460, 1415	O-H in-plane bending, C-H in-plane bending in OCH_3_ (lignin), C–C stretching of aromatic ring (lignin), symmetrical bending vibrations in CH_2_	lignin, cellulose, hemicellulose
3 *	1510	aromatic skeletal vibrations	lignin
4	1368	C–H, O–H bending vibrations	cellulose, hemicellulose
5	1320–1315	CH_2_ wagging	cellulose, hemicellulose
6	1244–1240	C–O stretch, O–H in plane vibrations	cellulose, hemicellulose
7	1153–1151	C–O–C asymmetric stretching vibrations	cellulose, hemicellulose
8	1096	aromatic C–H in-plane deformation, C–O–C asymmetric stretching vibrations	lignin, cellulose, hemicellulose
9	1030–1020	C-O stretching	lignin, hemicellulose
10	900–880	C-H vibrations of aromatic ring, C-H vibrations of polysaccharide units	lignin, cellulose, hemicellulose

**Table 2:** Characteristics connected with seed germination, seedling vigor, and R/S ratio of buckwheat and durum wheat after non-thermal plasma treatment with DCSBD and MSDBD at various treatment times. Statistical results of Tukey test (*p* < 0.05) were used; significant differences among treatments are given by different letters; positive trends are marked in bold.

Plants	Apparatus	Treatment Time (s)	Germination on the 3rd Day (%)	Germination on the 6th Day (%)	Germination Rate (%)	Germination Index	Seedling Vigor Index I. (mm)	Seedling Vigor Index II. (mg)	Seedling Vigor Index III. (mg)	R/S_Length	R/S_Fresh_ Weight	R/S_Dry_ Weight
			Mean ± SE	Mean ± SE	Mean ± SE	Mean ± SE	Mean ± SE	Mean ± SE	Mean ± SE	Mean ± SE	Mean ± SE	Mean ± SE
Buckwheat	control	0	44.0 ± 5.5 ab	44.0 ± 5.5 ab	**100 ± 0.0 a**	6.6 ± 0.8 ab	45.1 ± 7.3 ab	0.7 ± 0.2 ab	0.07 ± 0.01 ab	1.7 ± 0.1 a	0.2 ± 0.1 a	0.19 ± 0.03 a
	DCSBD	3	45.3 ± 5.9 ab	47.3 ± 6.5 ab	96.4 ± 2.6 a	6.9 ± 0.9 ab	46.0 ± 9.3 ab	0.6 ± 0.2 ab	0.07 ± 0.02 ab	1.8 ± 0.3 a	**0.3 ± 0.2 a**	0.14 ± 0.01 a
		5	52.7 ± 2.9 a	53.3 ± 3.2 a	98.9 ± 1.1 a	7.9 ± 0.4 a	49.3 ± 3.7 ab	0.7 ± 0.1 ab	0.09 ± 0.01 ab	1.6 ± 0.2 a	0.2 ± 0.0 a	0.19 ± 0.01 a
		10	59.3 ± 4.3 a	59.3 ± 4.4 a	**100 ± 0.0 a**	8.9 ± 0.6 a	**58.2 ± 3.8 b**	0.9 ± 0.1 b	0.09 ± 0.02 b	1.5 ± 0.2 a	0.2 ± 0.0 a	0.23 ± 0.02 a
		20	48.7 ± 1.7 ab	48.7 ± 1.8 ab	**100 ± 0.0 a**	7.3 ± 0.3 ab	34.9 ± 4.2 ad	0.7 ± 0.1 ab	0.07 ± 0.01 ab	1.1 ± 0.1 a	0.1 ± 0.0 a	0.19 ± 0.03 a
		30	34.0 ± 2.9 b	34.0 ± 2.10 b	**100 ± 0.0 a**	5.1 ± 0.4 b	19.9 ± 3.3 cd	0.4 ± 0.1 ac	0.03 ± 0.00 c	1.5 ± 0.2 a	0.2 ± 0.0 a	0.20 ± 0.03 a
		40	5.3 ±1.7 c	5.3 ±1.8 c	-	0.8 ± 0.3 c	2.1 ± 0.9 c	0.0 ± 0.0 c	0.00 ± 0.00 c	-	-	-
	control	0	44.0 ± 5.5 a	44.0 ± 5.5 a	**100 ± 0.0 a**	6.6 ± 0.8 a	45.1 ± 7.3 ab	0.7 ± 0.2 a	0.07 ± 0.01 a	1.7 ± 0.1 a	0.2 ± 0.1 a	0.19 ± 0.03 a
	MSDBD	3	54.7 ± 3.3 a	56.7 ± 3.8 a	96.7 ± 1.3 a	8.3 ± 0.5 a	47.6 ± 4.7 ab	0.9 ± 0.1 a	0.09 ± 0.02 a	1.8 ± 0.1 a	0.2 ± 0.0 a	0.16 ± 0.02 a
		5	**60.7 ± 6.0 a**	**60.7 ± 6.1 a**	**100 ± 0.0 a**	**9.1 ± 0.9 a**	55.7 ± 7.4 b	**1.1 ± 0.2 a**	**0.12 ± 0.02 a**	2.4 ± 0.3 ab	0.2 ± 0.1 a	0.21 ± 0.05 a
		10	58.7 ± 3.4 a	58.7 ± 3.5 a	**100 ± 0.0 a**	8.8 ± 0.5 a	41.4 ± 2.8 ab	1.0 ± 0.1 a	0.10 ± 0.01 a	**3.8 ± 0.1 c**	0.2 ± 0.1 a	0.25 ± 0.04 a
		20	44.7 ± 5.6 a	44.7 ± 5.7 a	**100 ± 0.0 a**	6.7 ± 0.8 a	32.9 ± 3.6 a	0.7 ± 0.1 a	0.06 ± 0.01 a	3.6 ± 0.2 bc	0.1 ± 0.0 a	0.19 ± 0.01 a
		30	52.7 ± 8.1 a	52.7 ± 8.2 a	**100 ± 0.0 a**	7.9 ± 1.2 a	31.6 ± 4.5 a	0.9 ± 0.3 a	0.09 ± 0.03 a	2.6 ± 0.5 ab	**0.3 ± 0.1 a**	0.23 ± 0.02 a
		40	49.3 ± 3.4 a	50.7 ± 3.7 a	97.6 ± 1.5 a	7.5 ± 0.5 a	38.2 ± 2.3 ab	0.8 ± 0.2 a	0.06 ± 0.01 a	1.7 ± 0.3 a	0.2 ± 0.0 a	**0.76 ± 0.58 a**
Hard wheat	control	0	88.0 ± 3.4 a	88.7 ± 3.4 a	99.3 ± 0.7 a	13.2 ± 0.5 a	147.7 ± 5.8 a	3.1 ± 0.2 a	0.37 ± 0.02 ab	1.0 ± 0.0 a	**1.1 ± 0.1 b**	1.00 ± 0.06 a
	DCSBD	3	87.3 ± 1.6 a	88.0 ± 1.7 a	99.3 ± 0.7 a	13.1 ± 0.2 a	135.8 ± 7.1 a	2.9 ± 0.1 a	0.36 ± 0.02 abc	1.0 ± 0.1 a	0.8 ± 0.0 a	0.86 ± 0.05 ab
		5	91.3 ± 2.3 a	91.3 ± 2.3 a	100.0 ± 0.0 a	13.7 ± 0.3 a	143.1 ± 5.6 a	3.1 ± 0.1 a	**0.42 ± 0.03 b**	**1.2 ± 0.0 a**	1.0 ± 0.1 ab	1.01 ± 0.05 a
		10	86.7 ± 2.8 a	86.7 ± 2.8 a	100.0 ± 0.0 a	13.0 ± 0.4 a	144.8 ± 6.3 a	2.7 ± 0.2 a	0.32 ± 0.02 ac	1.0 ± 0.1 a	0.8 ± 0.1 ab	0.79 ± 0.03 ab
		20	**92.0 ± 1.7 a**	**92.0 ± 1.7 a**	100.0 ± 0.0 a	**13.8 ± 0.3 a**	**155.8 ± 3.8 a**	2.9 ± 0.1 a	0.40 ± 0.02 ab	1.0 ± 0.1 a	0.7 ± 0.1 a	0.86 ± 0.05 ab
		30	83.3 ± 4.2 a	86.0 ± 4.0 a	**103.5 ± 3.5 a**	12.8 ± 0.6 a	138.3 ± 9.4 a	2.8 ± 0.3 a	0.28 ± 0.02 b	1.0 ± 0.1 a	0.8 ± 0.1 ab	0.63 ± 0.05 b
		40	87.3 ± 3.4 a	87.3 ± 3.4 a	100.0 ± 0.0 a	13.1 ± 0.5 a	144.3 ± 5.4 a	3.0 ± 0.2 a	0.34 ± 0.02 abc	1.0 ± 0.0 a	0.9 ± 0.1 ab	0.96 ± 0.10 a
	control	0	88.0 ± 3.4 a	88.7 ± 3.4 a	99.3 ± 0.7 a	13.2 ± 0.5 a	147.7 ± 5.8 b	3.1 ± 0.2 a	0.37 ± 0.02 a	1.0 ± 0.0 ab	**1.1 ± 0.1 a**	1.00 ± 0.06 a
	MSDBD	3	82.7 ± 4.4 a	83.3 ± 4.3 a	101.0 ± 1.6 a	12.5 ± 0.6 a	117.3 ± 15.2 b	2.6 ± 0.3 a	0.31 ± 0.04 a	0.9 ± 0.1 ab	1.0 ± 0.1 a	0.95 ± 0.09 a
		5	86.0 ± 3.6 a	86.7 ± 3.9 a	99.3 ± 0.7 a	12.9 ± 0.6 a	104.8 ± 7.7 ab	2.7 ± 0.3 a	0.34 ± 0.03 a	0.9 ± 0.1 a	0.9 ± 0.1 a	0.98 ± 0.04 a
		10	89.3 ± 2.7 a	91.3 ± 0.8 a	102.6 ± 3.6 a	13.6 ± 0.1 a	129.0 ± 8.0 ab	3.2 ± 0.1 a	0.37 ± 0.02 a	1.0 ± 0.1 ab	**1.1 ± 0.0 a**	**1.19 ± 0.03 a**
		20	80.0 ± 11.3 a	80.0 ± 11.3 a	100.0 ± 0.0 a	12.0 ± 1.7 a	99.8 ± 15.3 a	2.3 ± 0.4 a	0.26 ± 0.05 a	0.9 ± 0.0 ab	0.9 ± 0.1 a	0.93 ± 0.11 a
		30	90.0 ± 1.1 a	90.0 ± 1.1 a	100.0 ± 0.0 a	13.5 ± 0.2 a	130.0 ± 7.8 ab	**3.5 ± 0.3 a**	0.39 ± 0.01 a	1.0 ± 0.0 ab	0.9 ± 0.1 a	1.00 ± 0.03 a
		40	90.0 ± 2.4 a	90.1 ± 2.4 a	99.3 ± 0.7 a	13.5 ± 0.4 a	136.2 ± 5.1 ab	3.3 ± 0.3 a	0.35 ± 0.02 a	1.0 ± 0.0 b	**1.1 ± 0.1 a**	0.96 ± 0.03 a

**Table 3:** Characteristics connected with length and weight of seedlings of buckwheat and durum wheat after non-thermal plasma treatment with DCSBD and MSDBD at various treatment times. Statistical results of Tukey test (*p* < 0.05) were used; significant differences among treatments are given by different letters; positive trends are marked in bold.

Plants	Apparatus	Treatment Time (s)	Length of Root on the 3rd Day (mm)	Length of Root on the 6th Day (mm)	Length of Shoot (mm)	Length of Seedling (mm)	Weight of Fresh Root (mg)	Weight of Fresh Shoot (mg)	Weight of Fresh Seedling (mg)	Weight of Dried Root (mg)	Weight of Dried Shoot (mg)	Weight of Dried Seedling (mg)
			Mean ± SE	Mean ± SE	Mean ± SE	Mean ± SE	Mean ± SE	Mean ± SE	Mean ± SE	Mean ± SE	Mean ± SE	Mean ± SE
Buckwheat	control	0	24.1 ± 2.4 ab	63.3 ± 4.8 a	37.4 ± 3.5 a	100.7 ± 7.7 a	0.3 ± 0.1 b	1.4 ± 0.2 a	1.6 ± 0.3 a	**0.03 ± 0.01 a**	0.13 ± 0.01 ab	0.16 ± 0.02 a
	DCSBD	3	22.0 ± 3.1 ab	58.9 ± 4.0 a	36.1 ± 5.5 ab	95.0 ± 7.7 a	0.2 ± 0.0 ab	1.0 ± 0.2 a	1.2 ± 0.2 a	0.02 ± 0.00 a	0.13 ± 0.02 ab	0.14 ± 0.02 a
		5	24.0 ± 2.4 ab	55.7 ± 4.0 ac	36.5 ± 1.7 a	92.3 ± 3.0 a	0.2 ± 0.0 ab	1.2 ± 0.1 a	1.4 ± 0.1 a	**0.03 ± 0.00 a**	**0.14 ± 0.00 b**	0.16 ± 0.01 a
		10	**30.2 ± 0.7 b**	58.8 ± 2.7 a	**39.7 ± 3.7 a**	98.5 ± 2.8 a	0.2 ± 0.0 ab	1.3 ± 0.1 a	1.5 ± 0.1 a	**0.03 ± 0.00 a**	0.13 ± 0.02 ab	0.15 ± 0.02 a
		20	15.2 ± 0.9 ac	37.0 ± 4.9 bc	34.0 ± 2.4 ab	70.9 ± 6.4 a	0.2 ± 0.1 ab	1.2 ± 0.1 a	1.4 ± 0.2 a	0.00 ± 0.01 b	0.12 ± 0.01 ab	0.14 ± 0.01 a
		30	17.9 ± 2.3 ac	34.3 ± 3.5 b	24.0 ± 4.2 ab	58.3 ± 7.4 bc	0.2 ± 0.0 ab	0.9 ± 0.1 a	1.0 ± 0.1 a	0.02 ± 0.00 ab	0.08 ± 0.00 a	0.10 ± 0.01 a
		40	10.4 ± 4.2 c	13.7 ± 6.0 d	17.0 ± 6.8 b	30.8 ± 12.7 b	0.0 ± 0.0 a	0.1 ± 0.0 b	0.1 ± 0.0 b	0.00 ± 0.00 b	0.01 ± 0.00 c	0.01 ± 0.00 b
	control	0	24.1 ± 2.4 a	63.3 ± 4.8 a	37.4 ± 3.5 c	**100.7 ± 7.7 b**	0.3 ± 0.1 a	1.4 ± 0.2 a	1.6 ± 0.3 a	**0.03 ± 0.01 a**	0.13 ± 0.01 a	0.16 ± 0.02 a
	MSDBD	3	25.1 ± 2.0 a	54.3 ± 5.1 a	23.0 ± 1.9 ac	84.3 ± 6.7 ab	0.2 ± 0.1 a	1.3 ± 0.1 a	1.6 ± 0.1 a	0.02 ± 0.00 a	**0.14 ± 0.02 a**	0.16 ± 0.02 a
		5	26.0 ± 3.8 a	**64.1 ± 7.3 a**	27.4 ± 3.7 abc	91.6 ± 9.8 ab	**0.4 ± 0.1 a**	**1.5 ± 0.2 a**	**1.8 ± 0.2 a**	**0.03 ± 0.01 a**	0.12 ± 0.01 a	**0.18 ± 0.02 a**
		10	25.8 ± 1.8 a	56.5 ± 4.6 a	14.7 ± 1.0 b	71.2 ± 5.6 ab	0.3 ± 0.1 a	1.4 ± 0.1 a	1.7 ± 0.1 a	**0.03 ± 0.01 a**	**0.14 ± 0.01 a**	0.17 ± 0.01 a
		20	26.4 ± 1.9 a	58.8 ± 3.9 a	16.3 ± 1.0 ab	75.1 ± 4.7 ab	0.1 ± 0.0 a	1.3 ± 0.2 a	1.5 ± 0.2 a	0.02 ± 0.00 a	0.11 ± 0.01 a	0.13 ± 0.02 a
		30	18.5 ± 1.3 a	42.8 ± 4.4 a	20.2 ± 5.8 ab	63.0 ± 7.3 a	**0.4 ± 0.1 a**	1.3 ± 0.2 a	1.6 ± 0.3 a	**0.03 ± 0.01 a**	0.13 ± 0.02 a	0.16 ± 0.03 a
		40	20.6 ± 2.2 a	46.6 ± 4.6 a	29.3 ± 2.7 ac	75.9 ± 3.3 ab	0.3 ± 0.1 a	1.3 ± 0.2 a	1.5 ± 0.3 a	**0.03 ± 0.01 a**	0.09 ± 0.02 a	0.12 ± 0.01 a
Hard wheat	control	0	45.0 ± 2.9 a	84.4 ± 1.4 a	82.2 ± 1.7 a	166.6 ± 1.5 a	1.8 ± 0.1 a	1.7 ± 0.1 a	3.4 ± 0.2 a	0.21 ± 0.01 ab	0.21 ± 0.01 a	0.41 ± 0.01 ab
	DCSBD	3	43.7 ± 2.8 a	77.7 ± 5.9 a	76.5 ± 3.1 a	154.2 ± 7.3 a	1.4 ± 0.1 a	1.9 ± 0.1 a	3.3 ± 0.1 a	0.19 ± 0.01 ab	0.22 ± 0.01 a	0.41 ± 0.01 ab
		5	47.1 ± 2.4 a	83.7 ± 1.3 a	72.9 ± 3.4 a	156.6 ± 4.5 a	1.7 ± 0.1 a	1.7 ± 0.1 a	3.4 ± 0.2 a	**0.23 ± 0.01 b**	**0.23 ± 0.01 a**	**0.46 ± 0.02 b**
		10	**47.8 ± 1.8 a**	**86.6 ± 6.4 a**	80.9 ± 1.7 a	167.5 ± 7.7 a	1.4 ± 0.1 a	1.7 ± 0.1 a	3.1 ± 0.2 a	0.16 ± 0.01 ac	0.20 ± 0.01 a	0.36 ± 0.02 ac
		20	46.5 ± 0.7 a	85.9 ± 3.1 a	**83.5 ± 2.5 a**	**169.4 ± 2.7 a**	1.4 ± 0.1 a	1.8 ± 0.0 a	3.2 ± 0.1 a	0.20 ± 0.01 ab	**0.23 ± 0.00 a**	0.43 ± 0.01 ab
		30	42.9 ± 2.5 a	83.5 ± 6.1 a	82.2 ± 2.3 a	165.8 ± 7.1 a	1.5 ± 0.1 a	1.8 ± 0.1 a	3.4 ± 0.2 a	0.13 ± 0.01 c	0.21 ± 0.01 a	0.34 ± 0.01 b
		40	42.1 ± 1.9 a	84.7 ± 5.7 a	81.5 ± 3.5 a	166.2 ± 8.9 a	1.6 ± 0.1 a	1.8 ± 0.1 a	3.4 ± 0.1 a	0.19 ± 0.01 ab	0.20 ± 0.01 a	0.39 ± 0.02 ab
	control	0	45.0 ± 2.9 b	84.4 ± 1.4 b	82.2 ± 1.7 c	166.6 ± 1.5 b	1.8 ± 0.1 a	1.7 ± 0.1 ab	3.4 ± 0.2 ab	0.21 ± 0.01 ab	0.21 ± 0.01 a	0.41 ± 0.01 ab
	MSDBD	3	35.9 ± 3.3 ab	67.9 ± 7.8 ab	71.5 ± 5.3 ab	139.4 ± 12.9 ab	1.5 ± 0.2 a	1.6 ± 0.1 ab	3.4 ± 0.3 ab	0.18 ± 0.02 ab	0.19 ± 0.01 a	0.37 ± 0.03 ab
		5	33.1 ± 1.9 a	57.0 ± 3.5 a	63.9 ± 4.0 a	120.8 ± 5.9 a	1.5 ± 0.1 a	1.6 ± 0.1 ab	3.4 ± 0.2 ab	0.19 ± 0.01 ab	0.19 ± 0.01 a	0.38 ± 0.02 ab
		10	36.2 ± 1.1 ab	74.0 ± 2.6 ab	69.9 ± 3.6 ab	143.9 ± 5.5 ab	**1.9 ± 0.1 a**	1.7 ± 0.1 ab	3.6 ± 0.1 ab	**0.23 ± 0.01 a**	0.19 ± 0.01 a	0.42 ± 0.01 a
		20	31.4 ± 1.0 a	60.2 ± 4.3 a	64.5 ± 2.4 a	124.8 ± 6.6 a	1.3 ± 0.2 a	1.4 ± 0.2 a	2.7 ± 0.3 a	0.14 ± 0.01 b	0.16 ± 0.02 a	0.31 ± 0.03 b
		30	39.7 ± 2.0 ab	74.0 ± 4.3 ab	70.2 ± 4.4 ab	144.3 ± 8.4 ab	**1.9 ± 0.2 a**	**2.0 ± 0.1 b**	**3.9 ± 0.3 b**	0.22 ± 0.01 a	0.21 ± 0.00 a	0.43 ± 0.01 a
		40	39.4 ± 1.1 ab	80.0 ± 2.7 b	70.4 ± 2.2 ab	150.4 ± 4.8 ab	**1.9 ± 0.1 a**	1.7 ± 0.1 ab	3.6 ± 0.2 ab	0.19 ± 0.00 ab	0.20 ± 0.01 a	0.39 ± 0.02 ab

**Table 4:** Summary of the identified positive trends in the characteristics associated with the seed germination and early seedling growth of buckwheat and durum wheat after DCSBD and MSDBD plasma treatments (connection with Table 2 and Table 3).

	Treatment Time (s)	DCSBD	MSDBD	Suma
Buckwheat	3	1×	1×	2×
	5	2×	12×	14×
	10	5×	4×	9×
	20	1×	1×	2×
	30	1×	4×	5×
	40	0×	2×	2×
Sum		10×	24×	34×
Durum wheat	3	0×	0×	0×
	5	5×	0×	5×
	10	2×	4×	6×
	20	7×	0×	7×
	30	1×	4×	5×
	40	0×	2×	2×
Sum		15×	10×	25×

## Data Availability

All the data are included in the main text.
